# Implications of Severe Acute Respiratory Syndrome Coronavirus 2 (SARS-CoV-2) Infected Hospitalised Patients with Co-Infections and Clinical Outcomes

**DOI:** 10.3390/microorganisms11081921

**Published:** 2023-07-28

**Authors:** Jehad A. Aldali, Hamzah J. Aldali, Razan Aljohani, Mohammad Algahtani, Sultan Ayoub Meo, Saad Alharbi, Hani Al-Afghani, Linda Nazmi Aldabaseh, Elham Hamed Al Rubai, Abdulaziz Fallata, Saleh Abdullah Zahrani, Mohanad Atiah Al Zahrani

**Affiliations:** 1 Department of Pathology, College of Medicine, Imam Mohammad Ibn Saud Islamic University, Riyadh 13317, Saudi Arabia; jaaldali@imamu.edu.sa; 2 Cellular and Molecular Medicine, College of Biomedical Science, University of Bristol, Bristol City BS8 1QU, UK; 3 Hematology and Immunology, Faculty of Applied Medical Sciences, Tabuk University, Tabuk City 47512, Saudi Arabia; 4 Department of Laboratory and Blood Bank, Security Forces Hospital, Makkah 24251, Saudi Arabiahmalafghani@sfhm.med.sa (H.A.-A.); 5 Department of Physiology, College of Medicine, King Saud University, Riyadh 11461, Saudi Arabia; 6 Department of Laboratory, Comprehensive Specialized Clinics, Security Forces Hospital, Jeddah 11481, Saudi Arabia; 7 Department of Medicine, Security Forces Hospital, Makkah 24251, Saudi Arabia

**Keywords:** SARS-CoV-2, COVID-19, co-infections, hospital-acquired infections

## Abstract

The clinical severity of Severe Acute Respiratory Syndrome Coronavirus 2 (SARS-CoV-2) infection may rise because of acquiring a co-infection during the hospital stay of the patients. The rate of hospital co-infection alongside COVID-19 infection remains low. However, the mortality rates and intensive care unit (ICU) admission remains ambiguous. The present study investigates the implications of COVID-19 hospitalised infected patients with co-infection and the clinical outcomes. In this study, 142 patients were included. The eligible patients who tested positive for COVID-19 infection were hospitalised for more than two days. Each patient’s characteristics and laboratory results were collected, such as who was admitted to the intensive care unit and who was discharged or expired. The results revealed that out of the 142 hospitalised patients, 25 (17.6%) were co-infection positive, and 12 identified types of co-infection: two Gram-positive bacterial infections, one fungal infection and nine Gram-negative bacterial infections. In addition, 33 (23.2%) were ICU admitted, 21 were co-infection negative and 12 were co-infection positive. Among the 12 ICU admitted with co-infection, 33.4% were discharged. The death rate and ICU admission had a *p*-value < 0.05, indicating statistical significance for co-infected patients compared to non-co-infected patients. It was concluded that co-infection remains very low within hospitalised COVID-19-infected patients but can have severe outcomes with increased ICU admission and increased mortality rates. Thus, implementing infection preventive measures to minimize the spread of hospital-acquired infections among COVID-19 hospitalised patients.

## 1. Introduction

The Severe Acute Respiratory Syndrome Coronavirus 2 (SARS-CoV-2) has caused an outbreak of the COVID-19 pandemic, which began in December 2019 in Wuhan, China [1,2,3,4]. The SARS-CoV-2 infection has evolved into a worldwide health problem for public health systems in every country on Earth [5]. At the beginning of the pandemic, Zhu, and his colleagues [6], identified and characterized the virus that was preliminarily designated 2019-nCoV, then SARS-CoV-2, and finally COVID-19 [6]. The World Health Organization (WHO) was the first to assign the virus its current name, COVID-19 pandemic. They also announced in the year 2020 that the virus had spread internationally [5]. SARS-CoV-2 is a coronavirus, similar to the viral pathogens responsible for the 2002–2003 SARS pandemic and the 2012–2013 Middle East respiratory disease (MERS) outbreak. The coronaviruses contain a positive-sense single-stranded RNA genome and a helical capsid with a lipid bilayer envelope [6,7]. In terms of symptoms, spread, and timing, COVID-19 is quite similar to the influenza virus. The common symptoms include temperature, cough, headache, lack of smell, sore throat, and difficulty breathing. Moreover, some cases of COVID-19 in China ranged from having no clinical symptoms to having respiratory failure, septic shock, and/or multiple organ dysfunction [8]. Nevertheless, comorbid patients may require breathing apparatus and intensive care, increasing their susceptibility to secondary and opportunistic infections [9]. The clinical symptom variations in COVID-19 patients can be mediated by a variety of factors, which include, but are not restricted to, health conditions, age, gender, and other biological variables [10]. The reactivation of COVID-19 infection in a subpopulation of individuals following recovery from the first disease is a clinical phenomenon that has been discovered [11]. However, since the earliest detection of the COVID-19 infection, the incidence and mortality rates were extremely high compared to other flu infections. It is common to encounter a co-infection of COVID-19 and influenza viruses in many countries. Earlier studies showed that co-infections of bacteria and/or viruses have a direct association with more severe outcomes during a pandemic [12,13]. A study conducted in New York City indicated that the most common viral co-infection among hospitalised COVID-19-infected patients were rhinovirus, parainfluenza 3, influenza A and respiratory syncytial virus [14]. Respiratory viral co-infections can cause viral-induced airway damage by infecting epithelium cells and reducing mucociliary clearance. Systemic infectious viruses were also reported as co-infection with COVID-19, such as HIV, where two patients were required to have extensive care in the ICU with continuous monitoring on the mechanical ventilator [15]. The percentage of co-infections among hospitalised COVID-19-infected patients remains low, and hospitalised co-infected patients require more medical care and increased hospital stay. A study conducted in a public hospital in Brazil discovered that severe COVID-19 patients with secondary co-infections need longer admission and are at a greater likelihood of fatality [16]. Garcia-Vidal et al. [17] stated that at the time of COVID-19 diagnosis, community-acquired co-infection with COVID-19 was uncommon (3.1%) and was mostly caused by *Streptococcus pneumoniae* (*S. pneumonia*) and *Staphylococcus aureus* (*S. aureus*). In addition, hospital-acquired superinfections, with COVID-19, account for 4.7% of the total number of hospitalised COVID-19-infected patients, the majority of which were caused by *Pseudomonas aeruginosa* (*P. aeruginosa*) and *Escherichia coli* (*E. coli*) [17]. The prevalence rate of viral co-infection with COVID-19 has been previously reported up to 35.3%, and fungal and bacterial prevalence of co-infection up to 29.5% [18]. Goyal et al. [19] found a 6% risk of bacteremia during hospitalization, and Wang et al. [20] found a 42% rate of bacterial/fungal co-infection.

The clinical haematology laboratory plays an essential role by providing helpful prognostic indicators of disease and indicating bacterial infection or superinfection [21,22,23]. The presence of co-infections can change intestinal homeostasis, which can lead to infection triggers and further stimulate the immune cells to produce inflammatory markers such as cytokines (interleukin-1α (IL-1α) and IL-8) and chemokines [5,24]. In addition, it was previously found that mild co-infection with COVID-19 can increase a patient’s susceptibility to severe disease due to the impact of co-infection on the body’s immune function, especially in the elderly population [24]. Co-infections usually increase the levels of C-reactive protein (CRP) and procalcitonin (PCT) in the blood. Moreover, laboratory data of COVID-19-infected patients presented prolonged prothrombin time (PT), high lactate dehydrogenase (LDH), lymphopenia, increased alanine aminotransferase (ALT) and aspartate aminotransferase (AST), neutrophilia and increased D-dimer and troponin [5]. “PT-activated partial thromboplastin time (APTT), fibrinogen, antithrombin, fibrin degradation product (FDP), and D-dimer” were all assessed repeatedly over a two-week hospitalisation in a study of 183 patients with coronavirus pneumonia. The overall rate of mortality was 11.5%. On admission, non-survivors had considerably greater D-dimer and FDP levels, as well as longer PT and APTT times than survivors. During hospitalisation, fibrinogen and antithrombin levels were significantly reduced in non-survivors, while D-dimer and FDP levels were increased in all non-survivors, implying a “common coagulation activation, dysregulated thrombin generation, impaired natural anticoagulants, and fibrinolysis” [25]. However, the laboratory clinical results may vary depending on the type of co-infection.

To prevent the spread of co-infection of COVID-19 and other pathogens, it is established that implementing infection prevention measures will minimise co-infection cases. Additionally, early detection of co-infections is critical for identifying patients at high risk and determining the best therapies to prevent death [16]. It is best to avoid placing patients together in a shared room in the presence of seasonal flu infections. Isolating severely infected patients with continuous care and monitoring will mimic infection spread.

This retrospective study aims to characterise the consequences of co-infections in hospitalised patients based on their health state and pathogenic infection alongside COVID-19 in the ICU and non-ICU. The knowledge about COVID-19 co-infection situations has not been extensively studied. To properly treat patients and maintain sensible antimicrobial stewardship, it is critical to identify their pathogenic status in addition to COVID-19 infection. Therefore, this study hypothesises that increased mortality rates and ICU admission of hospitalised patients (≥48 h) are directly associated with co-infection alongside COVID-19 infection. Overall, this study sought to contribute to the understanding of the impact, outcomes, and potential effects of co-infections on hospitalised COVID-19-infected patients.

## 2. Subjects and Methods

This was a retrospective observational cohort study on patients who had been admitted to the hospital and were confirmed COVID-19 patients after 48 h of hospital admission between November 2020 to June 2021. To confirm accurate diagnoses of COVID-19 hospitalised patients, a polymerase chain reaction (PCR) test was conducted through nasopharyngeal throat swabs, which is a common specimen for respiratory infections. This method is considered a reliable method to detect the presence of COVID-19 in the collected specimen. Moreover, COVID-19-infected patients were also screened for co-infections while they were hospitalised. By screening for hospital co-infections, the research showed the impact of these infections in parallel with COVID-19 infections and the outcomes compared with hospitalised COVID-19 infections only. The main outcomes of interest were mortality rates and ICU admission, comparing COVID-19 infection only against co-infection with COVID-19. This study was designed without age restrictions; patients with all age groups were included in the study to allow a comprehensive assessment of the impact of co-infection in hospitalised patients with COVID-19.

### 2.1. Data Collection

In this retrospective cohort study, the data were collected from the Security Forces Hospital Makkah. The study involved collecting cohort-reported data of patients with ≥48 h of hospital stay. To prioritise patient privacy and protect the confidentiality of individual patients, the cohort data collected were anonymised during the collection and analysis process. The data were gathered from electronic medical records. The researchers collected demographic records such as age and gender. Data were also collected for ICU admission and whether a co-infection is present or not. If a co-infection was present, the type of infection was recorded. However, this was not always the case as some co-infections were not identified. The outcome assessed whether death or discharge from the hospital.

Furthermore, the researchers also collected the clinical laboratory results. These results include measurements and assessments of blood and disease markers for COVID-19-only infection and co-infected patients. The collected parameters were hypertension (HTN), chronic kidney disease (CKD), diabetes mellitus (DM), obesity, red blood cell (RBC) count, haemoglobin, prothrombin time (PT), partial thromboplastin time (APTT), D-dimer, C-reactive protein (CRP), ferritin, procalcitonin, neutropenia, thrombocytopenia, leukocytosis, creatine kinase (CK), troponin, lactate dehydrogenase (LDH), alanine transaminase (ALT), aspartate transferase (AST) and haemoglobin A1c (HBA1c).

To confirm the presence of co-infections, the microbiology lab used various microbiological tests, including cell culture, RT-PCR, antimicrobial susceptibility testing and biochemical. By employing these tests, the hospital would accurately validated the presence of co-infections among COVID-19 hospitalised patients. Inflammatory markers were collected during the study; however, they were not taken into consideration as a marker for co-infection. This was implemented to avoid introducing bias into the data analysis and results interpretation.

### 2.2. Data Analysis

Descriptive clinical and laboratory analysis was conducted to provide an overview of the effect of co-infection with hospitalised COVID-19 infection compared to non-co-infection, depending on the type of infection. Categorical and continuous data were presented as median and range alongside absolute numbers and percentages. The Mann–Whitney U test, a non-parametric test, was conducted to compare non-parametric data between the two groups. This test is suitable for a non-normally distributed variable. In contrast, the Chi-square test was utilized to compare frequencies between the groups. Both statistical tests were performed through GraphPad Prism V9.3.1 for Windows (GraphPad Software, San Diego, CA, USA) to compare co-infected patients with non-co-infection. A *p*-value < 0.05 was considered statistically significant.

### 2.3. Ethical Approval

The study was approved by the “Ethics Committee of Scientific Research, Security Forces Hospital, Makkah Al Mukarramah, Saudi Arabia” (Ref 0463-070222).

## 3. Results

In this study, 142 patients were included and met the eligibility criteria. The eligible patients that were tested positive for COVID-19 infection were hospitalised for more than 2 days. Patients were admitted to the hospital between November 2020 to June 2021. Among these participants, 52.8% were female with a median age of 59 ranging between 1.5 and 97 years old patients. The main characteristics of co-infected and non-co-infected patients are presented in Table 1. The data revealed the laboratory characteristic with significant differences between the co-infected and non-co-infected groups for CKD, hypertension, haemoglobin, the PT measure, prolactin, LDH and ALT enzyme. In contrast, the ferritin level, RBC count, thrombocytopenia, CK, PTT, troponin and HBA1C showed a non-significant statistical difference between the co-infected and non-co-infected groups. Moreover, ICU admission was statistically significantly observed between co-infected patients, with a total percentage of 48%, and those without, with only 17% of the total number of hospitalised COVID-19-only infected patients. Furthermore, the death rate also exhibited a statistically significant difference for co-infected patients (25 confirmed co-infected cases in total); the death rate was 32%. In contrast, there were only 117 hospitalised patients with only COVID-19 infection, and the death rate was only 3.4% (four cases).

The inflammatory markers were examined in both co-infected and non-co-infected COVID-19 hospitalised patients, including abnormal lymphocyte count, neutrophile, CRP, and D-dimer (Table 2). The statistical analysis demonstrated that there were significant differences in the inflammatory markers between the two groups. However, it is important to note that these markers were not taken into consideration as co-infection markers. Despite the observed differences, the median value of the leukocyte count in both groups is in the normal range. In contrast, the statistical test evaluated that among the co-infected group, CRP and D-dimer were significantly higher compared to those without co-infection.

At the hospital of Security Forces Hospital in Makkah Al Mukarramah, a total of 25 COVID-19 hospitalised patients were co-infection positive. Among these co-infected cases, only 12 individuals required ICU admission. The age, outcome and specific type of co-infection COVID-19 were documented in Table 3. The data revealed that a significant proportion of ICU-admitted patients did not survive, with approximately 66% of co-infected patients experiencing an unfavourable outcome. In addition, most ICU-admitted patients were above the age of 60, except for one patient.

**Figure 1:**
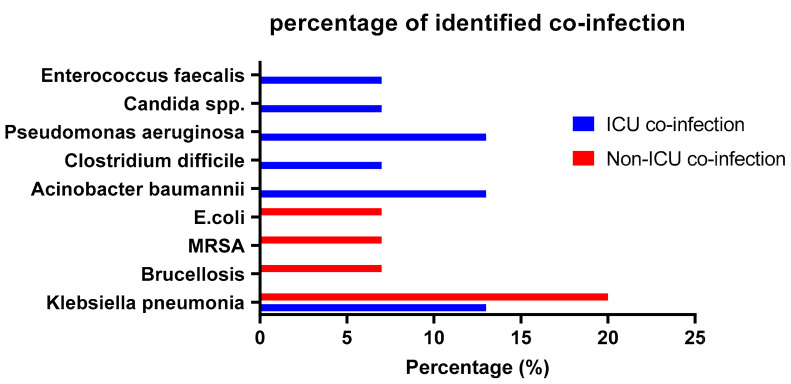
The frequency of hospital-acquired co-infection pathogens in COVID-19 patients. The figure shows the percentage of identified infections from co-infected patients in both the ICU and non-ICU admitted patients.

Gram-negative bacteria have been identified and isolated as the most common pathogens, with only two Gram-positives (*Staph aureus* and *Clostridium*) and one fungal infection (*Candida*). Co-infections from the 12 identified types included *Klebsiella pneumonia* with five cases, *Brucella* with one case, *E. coli* with one case, *Acinetobacter baumannii* with two cases, *Pseudomonas aeruginosa* with two cases, *Clostridium difficile* with one case, *Candida* spp. with one case, *Enterococcus faecalis* with one case, and Methicillin-resistant *Staph aureus* (MRSA) with one case (Figure 1).

## 4. Discussion

The SARS-CoV-2 infection has resulted in more than seven million deaths worldwide; however, co-infected patients are at a substantial risk of death [26]. The co-infection rates for COVID-19 infections are less than 8%, which is not as high as influenza co-infection [26]. Hospitalised patients who are infected with COVID-19 are at risk of encountering hospital-acquired infections [17]. Previous studies showed no significant influence of bacterial co-infection with COVID-19 if correct antimicrobial stewardship was applied [26]. However, this study was performed during the first wave of COVID-19 infection, and only a few patients were identified as having hospital-acquired co-infection in COVID-19 infected patients. The present study’s results reveal that 17.6% were co-infection positive, and 48% were admitted to the ICU, which shows that co-infection may have a serious impact on patient health.

Hospital-acquired Gram-negative infections are spreading worldwide, especially antibiotic-resistant strains [27]. The rate of bacterial co-infection with COVID-19 was higher compared to influenza, as well as the colonisation period [28]. Hospitalised patients encountering bacterial co-infected patients are at a greater risk of death by 2.7-fold compared to COVID-19-only infected patients. However, this is highly dependent on age and clinical complications, alongside the secondary infection. Respiratory viral infections disrupt airway epithelium and dysregulate the immune response, which is thought to promote bacterial colonisation.

Bacterial infections are widely recognised for their potential to complicate the clinical outcome of viral respiratory infections, such as influenza and COVID-19 infections. It was previously found that COVID-19 in Gram-negative co-infected patients is more prone to stay in the ICU than Gram-positive or fungal-infected patients [29]. Antibiotic-resistant Gram-negative bacteria are the predominant secondary infection in hospitalised COVID-19 patients, leading to immune complications [30]. The bacterial secondary co-infection can intensively influence the immune response, leading to a prolonged recovery period for hospitalised patients because of reduced immune clearance, tissue damage, respiratory distress and symptoms encountered through both COVID-19 and bacterial infection. In contrast, a previous report found that influenza A was one of the most common co-infections with hospitalised COVID-19 patients [31]. However, this can be selective to the location of the study and the endemic spread of influenza A. A systematic review performed between December 2019 and September 2020 found that the prevalence of influenza infection in patients with COVID-19 was approximately 1% [32]. Therefore, the recorded data from the Security Force Hospital, between November 2020 and June 2021, did not report any viral infection as a co-infection with COVID-19 hospitalised patients. Viral co-infection in hospitalised COVID-19 patients is rare compared to bacterial infection. However, respiratory syncytial virus is the most common secondary infection in COVID-19 patients. It is thought that the mediated immune response against COVID-19 infection in the respiratory tract reduces the risk of proliferation of the co-infected virus. Nevertheless, viral co-infection, as well as bacterial and fungal co-infection, can also increase the risk of mortality, morbidity, and clinical complications.

Studies conducted in Wuhan showed that ICU mortality rates and adverse outcomes are significant for patients with COVID-19 and secondary infection [33,34]. Furthermore, a recent study found that patients with community-acquired co-infection and hospital-acquired co-infections had worse outcomes compared to hospitalised patients to COVID-19 only [29]. Community-acquired bacterial infections, before hospitalization, were also associated with higher mortality risk rates, similar to hospital-acquired infections [26]. From our data, the mortality rate and ICU-admitted co-infected patients were highly significant compared to non-co-infected patients. This is consistent with the findings from four tertiary hospitals in Hong Kong from 2013 to 2017, which demonstrated that 5.6% (1087/19,361) of adult patients hospitalised for respiratory infections in a retrospective study had laboratory-confirmed viral-bacterial co-infection [35]. In comparison to patients with a viral infection, bacterial infection, or clinically suspected co-infection, these patients had considerably higher mortality rates. Similar to our findings, a greater rate of ICU admission was observed in patients who were co-infection positive. Co-infection is associated with increased in-hospital mortality, ICU admission and mechanical ventilation compared to hospitalised patients with only COVID-19 infection [26]. Additionally, 10,762 patients with community-acquired pneumonia were included in a meta-analysis of multiple studies, and it was discovered that co-infection results in a more challenging course with a greater need for mechanical ventilation and vasopressor medication. This confirms our findings, which indicated that higher ICU admission and fatality rates are related to viral-bacterial co-infection [36].

When the age group was investigated, many studies illustrated that elderly patients (>65 years of age) are at a higher risk for severe COVID-19 and/or any infectious disease [37]. The majority of cases (56.9% of all COVID-19 cases) involved people in their 18–49 s, whereas elderly patients over the age of 65 accounted for the most deaths, with 71% of all COVID-19 deaths. The elderly patient hospitalised with COVID-19 and influenza A H1N1 infection was admitted to the ICU and showed poor health symptoms [38]. The risk of ICU admission and mortality rates appeared to be high in the elderly population compared to patients below 60 years of age. These findings are reflected in our results as it was shown that eight out of 11 co-infected patients over the age of 65, who have been admitted to ICU, died. This accounted for about 72.72% of deaths of all co-infected elderly patients.

Our study investigated the characteristics of co-infected hospitalised patients with COVID-19 to identify the likelihood of ICU admission and mortality rates, which we found to be significant. Therefore, implementing correct infection prevention control (IPC) measures will minimise the risk of HAIs. In a surprising study at Santa Maria Hospital, with strict infection control awareness and staff training regarding IPC throughout the COVID-19 pandemic, Gram-negative nosocomial infections had risen by approximately 45% during the pandemic in 2020 compared to 2019 [39]. Contaminated clothing, gowns, and gloves with poor IPC measures during patient care can boost the transmission of bacterial and viral infections in patients [40,41,42]. On average, several studies found approximately 50% of nosocomial infections are the responsibility of contaminated hands of health workers [43]. A well-structured infection control program and the supported expertise of a hospital epidemiologist, statistician, and data manager are critical to the prevention of co-infections [44].

The literature also highlights that physical activity is important to modify the course of COVID-19 in a hospitalised population. Regular physical activity before becoming provides a sixty per cent chances of rapid recovery. Moreover, daily physical activity was linked to a milder course of SARS-CoV-2 infection, while poor physical activity was associated with a higher risk of death. Therefore, physicians must consider these physiological measures to fight against the COVID-19 pandemic [45].

Study strengths and limitations:

The strengths of this study compromise a reasonable number of hospitalised COVID-19-infected patients with various age groups and well-characterized laboratory data. In addition, the death rate and ICU-admitted patients were analysed. However, there are a few limitations to our study that must be acknowledged. Firstly, out of the 25 co-infected patients, 13 had an unidentified type of infection. The second limitation is that this study was only performed in one hospital, meaning hospital-acquired co-infection outcomes may vary depending on the type of infection. Finally, the data collected did not include any viral co-infection to analyse the potential severity of COVID-19 alongside the vaccination profile of patients.

## 5. Conclusions

The incidences of co-infection in hospitalised patients with COVID-19 remain low. Nevertheless, if co-infection is found, the risk of ICU admission and mortality rate may be increased, leading to worse clinical outcomes. The majority of reported cases were Gram-negative bacterial infections. Therefore, the correct implementation of antibiotic treatments and IPC measures will prevent and/or limit the spread of co-infection within hospitalised COVID-19-infected patients.

## Figures and Tables

**Table 1:** Comparison of demographics and main characteristics of COVID-19 patients with or without co-infection for ≥48 h of hospital admission.

Characteristic	All Patients (*n* = 142) N (%)	Without Co-Infections (*n* = 117) N (%)	With Co-Infections (*n* = 25) N (%)	*p* Value (Chi-Square/Mann-Whitney U Test)
Male Female	67 (47.8%) 75 (52.8%)	53 (45.3%) 64 (54.7%)	14 (65%) 11 (44%)	0.33
Age	59 (range 1.5–97)	56 (range 1.5–87)	70 (range 16–97)	0.002 (Mann Whitney)
CKD	17 (12%)	10 (8.5%)	7 (28%)	<0.01
Ferritin > 300 n/mL	66 (46%)	53 (45.3%)	13 (52%)	0.54
Hypertension mmHg	61(43%)	44 (37.6%)	17 (68%)	0.005
Diabetes mellitus	65(45.7%)	62 (53%)	13 (52%)	0.93
ICU admission	33 (23.2%)	21 (17.9%)	12 (48%)	0.001
Obesity	88 (62%)	77 (65.8%)	11 (44%)	0.04
RBCs > 6.1 × 10^6^/mL	4 (2.8%)	3 (2.6%)	1 (4%)	0.69
Hb < 12 g/100 mL	53 (37%)	36 (30.8%)	17 (68%)	<0.001
Thrombocytopenia < 150	21 (14.8%)	19 (16.2%)	2 (8%)	0.29
PT > 13.5 s	80 (56%)	59 (50.4%)	21 (84%)	0.002
PTT > 70 s	4 (2.8%)	2 (1.7%)	2 (8%)	0.08
Procalcitonin > 0.25 ng/mL	31 (21.8%)	15 (12.8%)	16 (64%)	<0.001
CK > 198 mmol/L	24 (17%)	17 (14.5%)	7 (28%)	0.1
Troponin > 0.04	7 (4.9%)	5 (4.3%)	2 (8%)	0.61
LDH > 333	35(24.6%)	24 (20.5%)	11 (44%)	0.01
ALT > 55	10 (7%)	5 (4.3%)	5 (20%)	0.005
AST > 33	42 (29.5%)	31 (26.5%)	11 (44%)	0.08
HBA1C > 5.7 mmol/L	60 (42%)	51 (43.6%)	9 (36%)	0.49
No. Death	12 (8.4%)	4 (3.4%)	8 (32%)	<0.0001

CKD: Chronic kidney disease, PT: Prothrombin time, RBCs: Red Blood Cells, PTT: Partial thromboplastin time, LDH: Lactate dehydrogenase, CK: Creatinine Kinase, ALT: Alanine transaminase, AST: Aspartate aminotransferase, HBAC1: Measures the amount of blood sugar (glucose) attached to the haemoglobin.

**Table 2:** Inflammatory indicators in COVID-19 patients with or without co-infections.

Characteristic	All Patients (*n* = 142)	Without Infections (*n* = 117)	With Infections (*n* = 25)	*p* Value (Mann-Whitney U Test)
Leukocyte (leukocyte/mL)	6.39 (4.5)	6.11 (3.6)	8 (6.1)	0.006
Neutrophil (neutrophil/mL)	4.4 (4)	3.9 (3.61)	5.7 (4.7)	0.003
CRP (mg/L)	4.49 (10.5)	4.09 (9.03)	12.14 (21.8)	0.003
D-dimer (mg/L)	0.8 (0.95)	0.7 (0.7)	1.7 (2)	<0.001

Data presented as median (IQR = interquartile range), CRP: C-reactive protein.

**Table 3:** Distribution of COVID-19 patients in the ICU with co-infection. The table presents the number of patients, age, death/discharge, and the type of co-infection.

ICU Admission	P1	P2	P3	P4	P5	P6	P7	P8	P9	P10	P11	P12
Age	79	71	69	71	79	70	66	89	36	61	73	84
Death/discharge.	X	X	O	X	X	O	X	X	O	X	O	X
Pseudomonas	√				√							
Acetobacter Baumann		√									√	
Klebsiella pneumonia		√										√
Enterococcus faecalis			√									
Clostridium difficult												√
Candida					√			√				
Bacteremia		√			√	√						
Pneumonia						√			√	√		
Unspecified co-infection				√			√					

X = Death; O = Discharge; √ = Co-infection type.

## Data Availability

Data may be provided on reasonable request to corresponding author.
